# Network Pharmacology-Based Approach to Investigate the Mechanisms of Mahai Capsules in the Treatment of Cardiovascular Diseases

**DOI:** 10.1155/2020/9180982

**Published:** 2020-05-12

**Authors:** Minjuan Shi, Bo Li, Qiuzhen Yuan, Xuefeng Gan, Xiao Ren, Shanshan Jiang, Zhuo Liu

**Affiliations:** ^1^Department of Pharmacy, Shaanxi Provincial Hospital of Traditional Chinese Medicine, Xi'an 710000, Shaanxi, China; ^2^Department of Pharmacy, Xi'an Children's Hospital, Xi'an 710000, Shaanxi, China

## Abstract

**Background:**

Mahai capsules (MHC) have been deemed to be an effective herb combination for treatment of cardiovascular diseases (CVD) development and improvement of the life quality of CVD patients. To systematically explore the mechanisms of MHC in CVD, a network pharmacology approach mainly comprising target prediction, network construction, biological process and pathway analysis, and related diseases was adopted in this study.

**Methods:**

We collected the bioactive compounds and potential targets of MHC through the TCMSP servers. Candidate targets related to CVD were collected from Therapeutic Targets Database and PharmGkb database and analyzed using ClueGO plugin in Cytoscape. KEGG pathway was enriched and analyzed through the EnrichR platform, and protein-protein interaction networks were calculated by STRING platform. The compound-target, target-disease, and compound-target-disease networks were constructed using Cytoscape.

**Results:**

A total of 303 targets of the 57 active ingredients in MHC were obtained. The network analysis showed that PTGS2, PTGS1, HSP90, Scn1a, estrogen receptor, calmodulin, and thrombin were identified as key targets of MHC in the treatment of CVD. The functional enrichment analysis indicated that MHC probably produced the therapeutic effects against CVD by synergistically regulating many biological pathways, such as PI3K-Akt, TNF, HIF-1, FoxO, apoptosis, calcium, T-cell receptor, VEGF, and NF-kappa *B* signaling pathway.

**Conclusions:**

In summary, the analysis of the complete profile of the pharmacological properties, as well as the elucidation of targets, networks, and pathways, can further illuminate that the underlying mechanisms of MHC in CVD might be strongly associated with its synergic regulation of inflammation, apoptosis, and immune function, and provide new clues for its future development of therapeutic strategies and basic research.

## 1. Background

Cardiovascular diseases (CVD) are a class of the degenerative chronic diseases such as atherosclerosis, heart failure, hypertensive, aneurysms, and thromboembolism [1]. CVD markedly impairs the quality of life of patients and has been the leading cause for morbidity and mortality. It has been reported that CVD deprives more than 10 million human lives each year, and the mortality is projected to be 23.6 million in 2030 [2]. The prevention and treatment of cardiovascular medicine have been dramatically progressed in the past years. Currently, the major pharmacologic options for CVD include angiotensin-converting enzyme inhibitors, sodium channel blockers, nitrate esters, and various thrombolytic agents [3, 4]. However, as a result of the complicated pathogenesis involved in CVD, single targeted therapies may not be sufficient and several certain inevitable side effects still exist. The medical failures of some patients with CVD might be due to the incomplete understanding of the complex underlying pathophysiology. With the enormous development of medical science, researchers gradually found that most diseases are usually caused by multiple targets instead of single gene. Hence, multicomponent drugs represented by traditional Chinese medicine (TCM), which had been widely used in health maintenance, have drawn increasing attention in CVD treatment [5]. TCM is a whole medical system with rich practice experience for thousands of years and has attracted a lot of attention in recent years because of valid treatment effects and fewer adverse reactions. The treatment of complex diseases using TCM has been considered as a complexity whole that confronts another whole, and it focuses on the state of the whole organisms by regulating all the elements within the body [6]. Based on the characteristics of multi-ingredients and multitargets feature, TCM treatment has enormous potential in treating chronic complex diseases including CVD.

In China, various TCM have achieved great success in the prevention and treatment of CVD. Danshen dripping pills and Danhong injection are notable examples that were confirmed by clinical trials in their protective effects against CVD [7–9]. Similarly, Mahai capsules (MHC) have been deemed to be a crucial strategy for treatment of CVD development and improvement of the life quality of CVD patients. MHC is an effective herb combination that consists of Hedysarum Multijugum Maxim (Huangqi, HQ), Strychni Semen (Maqianzi, MQ), Angelicae Sinensis Radix (Danggui, DG), Caulis Piperis Kadsurae (Haifengteng, HFT), Homalomena Occulta (Lour.) Schott (Qiannianjian, QN), and Radix Rhei Et Rhizome (Dahuang, DH). This formula is applied to inhibit CVD developments such as atherosclerosis, stroke, deep vein thrombosis, and cerebral infarction. An increasing number of clinical researches emphasize the positive effect of HQ in treating CVD, for example, HQ, has been proven to protect cardiovascular disease through multiple mechanisms, including anti-inflammatory and lipid lowering effects [10–12]. Several recent clinical studies showed that HQ can enhance myocardial contractility and myocardial cell excitation-contraction coupling and generate significant cardiotonic effect with the treatment of acute myocardial infarction [13, 14]. Besides, the efficacy and safety of HQ preparation in control of heart failure from cardiac dysfunction and metabolic alterations have also been proven [10]. DG has been widely used to treat blood deficiency disease in China, and the ameliorative effect of DG against heart injury and myocardial infarction has been studied [15, 16]. However, the complex active ingredients and underlying mechanisms of MHC on CVD have not been identified, and it complicates the modernization and clinical usage of MHC. Thus, it is necessary to identify the bioactive substances of MHC and understand their synergistic actions in and the exact effects on multiple targets.

Even though there was considerable benefit with such multicomponent drugs, understanding the scientific material basis and underlying mechanism of TCM herbal formulas are still required in the treatment of CVD at the molecular level and from a systematic perspective. Most herbal medicines containing enormous bioactive compounds and lots of related multiple targets also complicate the pharmacological research, making it difficult to clarify their pharmacological mechanism only by traditional experimental approaches. With the rapid development of life science and computer science, various virtual screening tools and bioinformatic database have been developed to explore the interactions between the complex ingredients and the huge amount of target genes in TCM [17, 18]. The network pharmacology-based approach provides guidance to investigate potential pharmacological actions and clarify complex molecular mechanisms of TCM [19]. Traditional Chinese Medicine Systems Pharmacology (TCMSP), PharmMapper, and many databases have been developed to predict the validated and potential targets [20, 21]. Meanwhile, there are several bioinformatic software and servers to analysis the biological and mechanical properties of compound targets, such as David, String, EnrichR, and Cytoscape.

In this paper, we aim to employ virtual screening databases and integrate bioinformatics analyses to explore the relationships between MHC and targets, which show the network of drug and related targets on whole level. This might enable us to investigate the pharmacological mechanism of how MHC exerts the protective effects on CVD.

## 2. Methods

### 2.1. Screening of Active Components and Pharmacokinetic Absorption, Distribution, Metabolism, and Excretion (ADME) Evaluation

Candidate compounds for Hedysarum Multijugum Maxim (Huangqi, HQ), Strychni Semen (Maqianzi, MQ), Angelicae Sinensis Radix (Danggui, DG), Caulis Piperis Kadsurae (Haifengteng, HF), Homalomena Occulta (Lour.) Schott (Qiannianjian, QN), and Radix Rhei Et Rhizome (Dahuang, DH) were retrieved from the TCMSP database (ref.). Identification of ADME (absorption, distribution, metabolism, and excretion) properties by the TCMSP database was employed to screen the composite compounds. In the current study, OB (prediction of oral bioavailability) and DL (prediction of drug-likeness) identify the potential bioactive compounds of MHC. The ingredients satisfying the criteria of OB ≥30% and DL ≥0.18 are retained and treated as candidate molecules of MHC for subsequent analysis.

### 2.2. Identification of Candidate Compounds and Target Genes

To gather information on interactions between active functional compounds in MHC and associated genes, the bioactive compounds and potential targets were collected through the TCMSP servers. Known CVD-related targets were collected from existing resources, including Therapeutic Targets Database and PharmGkb database, which provide comprehensive information on bioactive compounds and targets interactions, and relationships among compounds, targets, and diseases. All obtained proteins were subjected to PharmGkb or TTD to detect the relationships between candidate molecules, targets, and CVD.

### 2.3. Network Construction and Analysis

To investigate relationship between the compounds in MHC and their targets in CVD, we construct the network through network visualization software in Cytoscape 3.6.1. This software was used to integrate data and analysis and visualize complex interaction networks. In networks, nodes represent compounds or proteins and edges indicate compound-target gene interactions. Three types of networks, for example, compounds-targets (C-T), targets-diseases (T-D), and compounds-targets-diseases (C-T-D) networks, were constructed and visualized using Cytoscape. For nodes in the complex network, three indicators were calculated to reveal its features.

### 2.4. Gene Ontology (GO) Analysis

ClueGO, which is a plugin integrated in Cytoscape, can be employed to conduct GO analysis to create functionally organized term networks and comprehensively visualize functionally grouped terms for understanding the biological significances [22]. The *p* value was used to examine the significance of the GO terms enrichment. The GO terms that have a *p* value of ≤0.05 were regarded as significant and interesting.

### 2.5. Kyoto Encyclopedia of Genes and Genomes (KEGG) Pathway Analysis

Enrichr is an integrative web-based platform that includes new gene-set libraries, an alternative approach to rank enriched terms, and various interactive visualization approaches to display enrichment results [23]. The KEGG pathway was enriched and ranked based upon the combined score which is calculated by the EnrichR platform. An adjusted *p* value threshold of 0.05 was used for pathway discovery. In this study, we chose the top ten KEGG terms to explore the related pathways.

### 2.6. Protein-Protein Interaction (PPI) Networks

String (https://string-db.org/) was employed to construct PPI networks with the species limited to “homo sapiens” [24]. String is a known platform and forecasts the interactions of proteins, and it defines PPI with confidence ranges for data scores.

## 3. Results

### 3.1. The Candidate Compounds and Putative Target Proteins

Using in silico prescreening models, the main components of MHC with favorable pharmacokinetic characteristics were determined. From the 444 native MHC compounds collected from the TCMSP database, 75 candidate compounds were screened from the 6 herbs by ADME and prepared for further study as the candidate compounds as shown in Table 1.

**Figure 1:**
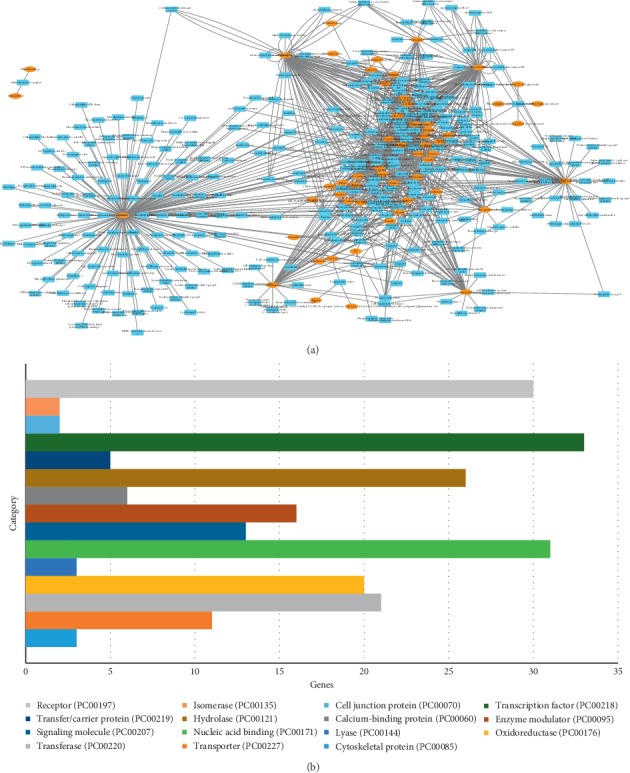
The C-T network of MHC and the targets class. (a) The compound in MHC and the potential target network. Different colors represent the nodes with different attributions. Yellow nodes represent the candidate compounds; blue represent the predicted targets. (b) The distribution of the candidate targets.

We compared the 303 putative target proteins for commonality and properties, and the results are shown in Figure 1(a). The distribution of the biochemical classification indicates that the target space mainly consists of transcription factor, receptor, nucleic acid binding, hydrolase, oxidoreductase, transferase, enzyme modulator, transporter, and signaling molecule. Remarkably, the obtained drug targets are enriched in transcription factor (15.3%), receptor (14.4%), and nucleic acid binding (14%), highlighting the critical roles of targets in drug discovery. Among the targets, 32 targets are receptors, 21 are transferases, and 14 are transporters (Figure 1(b)).

### 3.2. C-T Network Analysis

To uncover the synergistic effects of multicomponents and multitargets in MHC, a global view of the C-T network was generated. After removing 18 compounds with no target proteins, a graph of C-T interactions (Figure 1(a)) was constructed using 57 candidate compounds and their 303 potential targets. The average number of targets per compound is 5.3, showing the multitarget features and polypharmacology properties of constituents in MHC. The C-T network contains 360 nodes and 1056 ligand-target interactions. For most active compounds, quercetin, beta-sitosterol, and stigmasterol have high degree distributions, and each of them hits more than 90 potential targets. For instance, quercetin has the highest degree (154), followed by beta-sitosterol with 114 drug-target interactions and stigmasterol possessing 93 target proteins.

By further observation of the C-T network, we found that many targets are hit by different numbers of compounds, implying the multicomponent characteristics of herbs. Among these targets, PTGS2 possesses the largest degree (degree = 40), followed by PTGS1 (degree = 31), HSP90 (degree = 31), Scn1a (degree = 28), estrogen receptor (degree = 16), calmodulin (degree = 16), and thrombin (degree = 15), demonstrating their potential therapeutic effects for treating CVD. Among them, the target prostaglandin G/H synthase 2 (PTGS2) with the highest degree (40) and prostaglandin G/H synthase 1 (PTGS1) with a degree of 31 can be modulated by the compounds in MHC, indicating their vital role in helping to treat CVDs. Similarly, heat shock protein 90 (HSP90), a key enzyme in the blood coagulation system, was predicted to be regulated by 31 chemicals. The details of the relationship between activate compounds and targets are described in Table S1. All these results imply the probably different binding properties of active chemicals in MHC with the active substances and suggest that individual compounds may act on the same targets synergistically, thus exerting therapeutic effects on CVDs.

### 3.3. T-D Network and C-T-D Network Analysis

**Figure 2:**
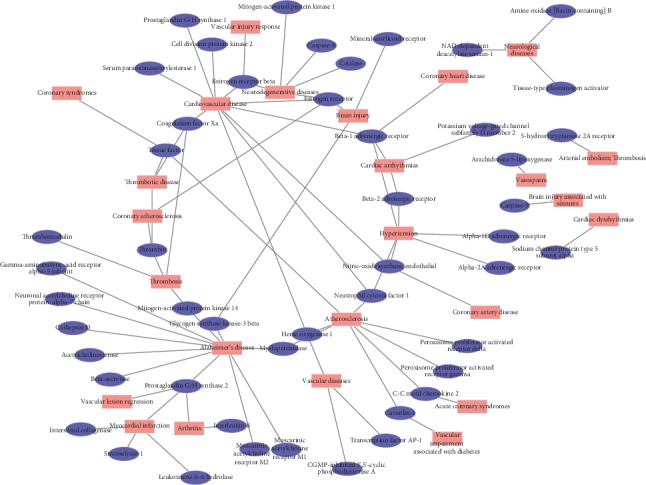
T-D network: a potential targets-CVD network and nodes represent targets and diseases.

To gain better insight into the diseases that could be modified by MHC, a T-D network was constructed on the strength of predicted targets and the corresponding diseases (Figure 2, Table S2). The gene entries related to CVD were collected from the TTD and PharmGkb database and converted into UniProtKB IDs to determine the correlation between the putative target proteins and CVD. Targets with the most degrees among the 49 targets were as follows: thrombin, prostaglandin G/H synthase 2, eNOS, estrogen receptor, and *β*-adrenergic receptor; the top diseases that were most relevant to MHC were as follows: atherosclerosis, cardiovascular disease, hypertension, thrombosis, and neurodegenerative diseases, implying that MHC may be also effective in the treatment of these diseases. Thrombin, a main target of MHC, was associated with coronary atherosclerosis, thrombosis, and thrombotic disease in the T-D network. Based on these findings, MHC contained numerous effective substances with different pharmacologic properties that may act on multiple targets with potential synergistic effects.

**Figure 3:**
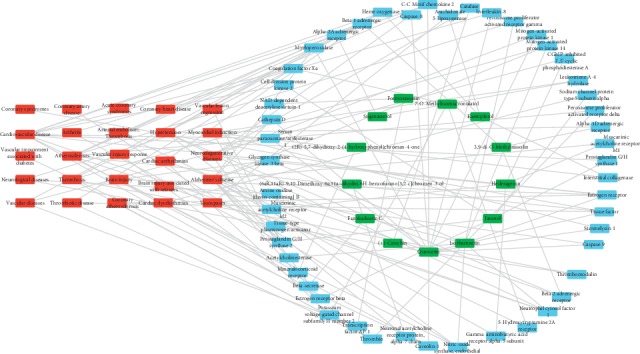
C-T-D network: a compounds-targets-CVD network and nodes represent compounds and targets.

Then, we mapped all candidate compounds with their corresponding targets onto these diseases. After discarding the targets without participating in any related CVDS and the corresponding compounds, a compounds-targets-diseases network was constructed with 87 nodes (13 compounds, 49 targets, and 25 diseases) and 127 edges (Figure 3). For instance, 3 compounds such as quercetin, isorhamnetin, and kaempferol are referred to as regulating important targets in atherosclerosis; isorhamnetin, 3,9-di-O-methylnissolin, and stigmasterol are referred to as regulating main targets in hypertension; quercetin, Jaranol isorhamnetin, and (+)-catechin are referred to as regulating key targets in neurodegenerative diseases. Our results suggest that different ingredients of MHC may be involved in different diseases.

### 3.4. GO Enrichment Analysis

**Figure 4:**
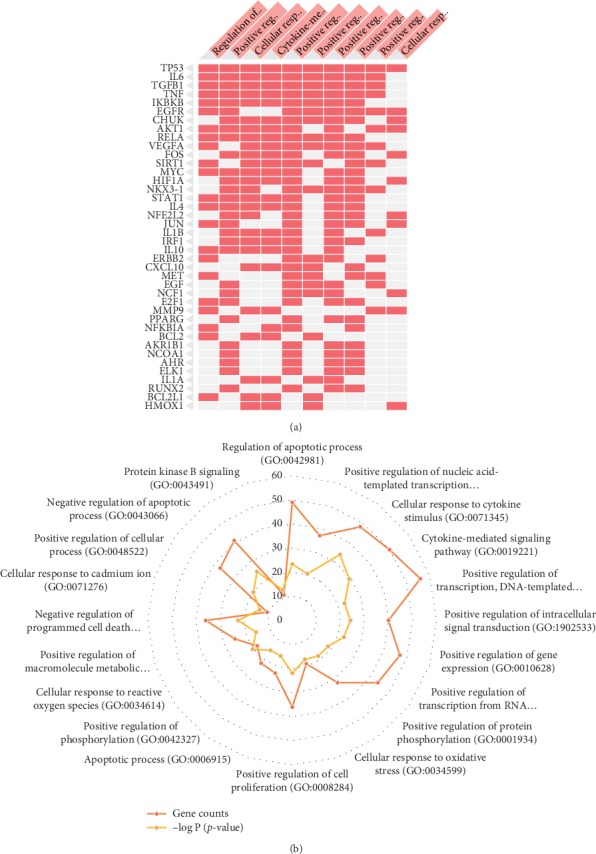
The GO BP analysis of predicted targets of MHC. EnrichR analysis was performed to identify the most significantly enriched GO BP terms.

To clarify the multiple mechanisms of MHC on CVD from a systematic level, we performed an enrichment analysis for the biological process (BP), molecular function (MF), and cellular component (CC) of the retrieved protein targets of MHC. As shown in Figure 4, the significantly enriched BP terms were mainly involved in regulation of apoptotic process, positive regulation of nucleic acid-templated transcription, cellular response to cytokine stimulus, cytokine-mediated signaling pathway, positive regulation of transcription, DNA-templated positive regulation of intracellular signal transduction, positive regulation of gene expression, positive regulation of transcription from RNA polymerase II promoter, and positive regulation of protein phosphorylation. The most frequently occurring protein targets were TP53, IL-6, TGFB1, TNF, IKBKB, EGFR, CHUK, AKT1, RELLA, VEGFA, FOS, SIRT1, MYC, HIF1A, NKX3-1, STAT1, and IL-4.

**Figure 5:**
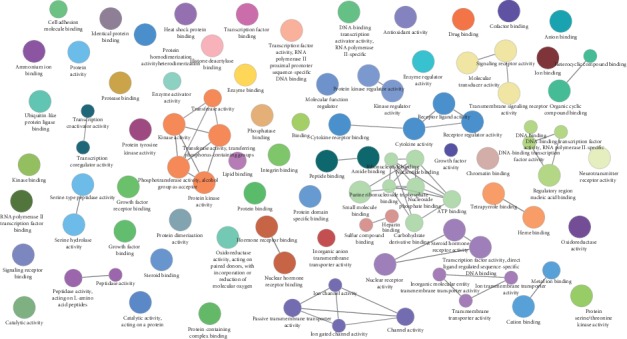
The GO MF analysis of predicted targets of MHC. ClueGO was used to identify the most significantly enriched GO MF terms.

**Figure 6:**
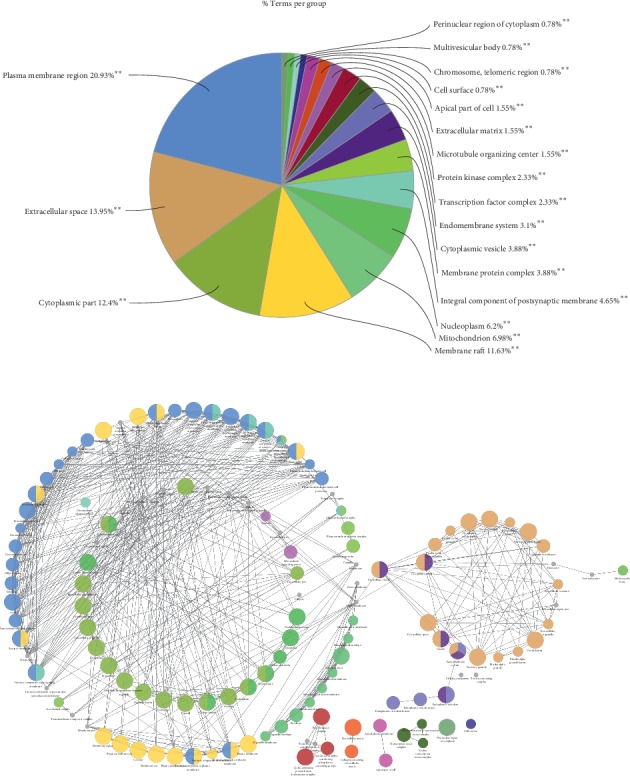
The GO CC analysis of predicted targets of MHC. ClueGO was used to identify the most significantly enriched GO CC terms.

Figure 5 lists the significantly enriched MF terms of these targets. The results suggested that targets of MHC were strongly correlated with the molecular functions such as protein binding, enzyme binding, receptor binding, identical protein binding, protein dimerization activity, G-protein coupled amine receptor activity, signal transducer activity, binding, molecular transducer activity, protein heterodimerization activity, steroid hormone receptor activity, drug binding, and signaling receptor activity. As shown in Figure 6, the top five cellular components were plasma membrane region (20.93%), extracellular space (13.95%), cytoplasmic part (12.4%), and membrane raft (11.63%). These abovementioned observations are valued in improved understanding of the mechanism of MHC.

### 3.5. Pathway Enrichment Analysis

**Figure 7:**
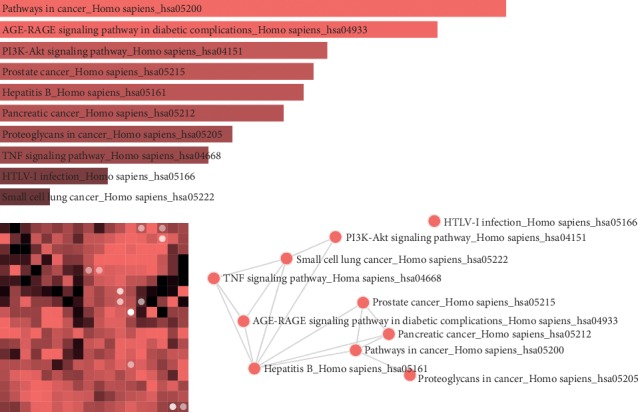
The KEGG enrichment analysis of predicted targets of MHC.

To investigate the underlying mechanism of MHC, the targets were further mapped to pathways, and the top 10 pathways are listed in Figure 7. Among the 177 enriched pathways, several pathways have been verified as important and accurate target pathways for curing CVDs, such as AGE-RAGE signaling pathway in diabetic complications (hsa04933), PI3K-Akt signaling pathway (hsa04151), TNF signaling pathway (hsa04668), HIF-1 signaling pathway (hsa04066), FoxO signaling pathway (hsa04068), apoptosis (hsa04210), calcium signaling pathway (hsa04020), T-cell receptor signaling pathway (hsa04660), MAPK signaling pathway (hsa04010), Toll-like receptor signaling pathway (hsa04620), focal adhesion (hsa04510), NOD-like receptor signaling pathway (hsa04621), VEGF signaling pathway (hsa04370), and NF-kappa *B* signaling pathway (hsa04064). Among them, the PI3K-Akt and TNF signaling pathways have the highest combined scores, which imply the vital roles in the treatment and prevention of CVDs. In addition, 6 signaling pathways including HIF-1, FoxO, VEGF, TLR, MAPK, and NF-*κ*B signaling pathways are also important pathways capable of regulating anti-inflammatory, neuroprotective, and antioxidative effects. This suggests that the potential targets of MHC may be involved in various pathways, showing their specific mechanism of action to modulate CVDs.

### 3.6. Protein-Protein Interactions

**Figure 8:**
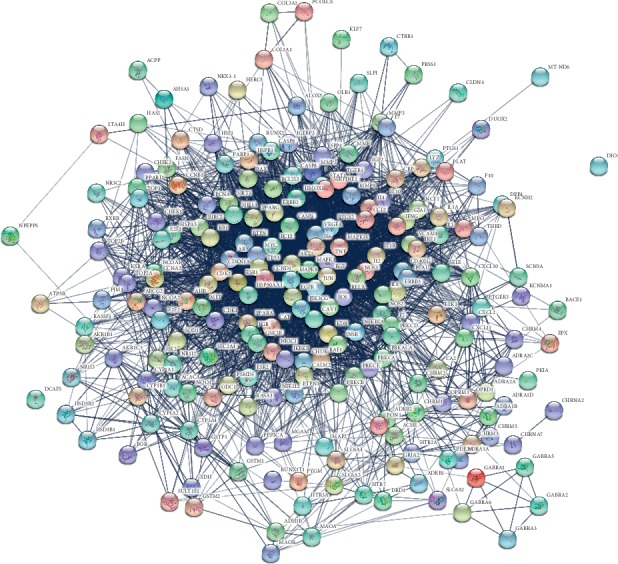
Network pharmacology analysis through the protein interaction of predicted protein targets of MHC. The network nodes were predicted proteins and the edges represented the functional associations.

The protein-protein network was constructed via mapping the putative targets into the String platform. After excluding isolated nodes, the protein interaction network induced by MHC was composed of 221 nodes (proteins) and 3865 edges (Figure 8). The topological properties of the network rewired by the MHC were analyzed with the network analyzer plugin. Among these properties, the node degree can be used to distinguish between random and scale-free network topologies. Three topological features of each node in the network were calculated to find the major nodes. Finally, 22 nodes were selected as major nodes, namely, TP53, JUN, AKT1, IL6, TNF, VEGFA, EGF, MAPK1, FOS, PIK3CG, MYC, BCL2, ESR1, EGFR, MAPK8, IL8, PTGS2, CASP3, HSP90AA1, MMP9, NOS3, and CCND1. Thus, these targets were likely to be the key or central proteins that MHC may directly act on them to treat CVDs.

## 4. Discussion

The efficacy of MHC has been verified through accumulated considerable clinical experiences. However, it is difficult to illuminate the mechanism of MHC from the perspective of modern medicine because of the complex composition. The network pharmacological analysis provides new approaches and perspectives for the study of complicated Chinese medicine formula. In the present study, we used the network pharmacology approach to illuminate the scientific material basis and multiple underlying mechanisms of MHC in CVDs treatment from a systematic perspective. The pharmacodynamic compounds and potential targets, network analysis of elements such as active ingredients and potential targets, GO and KEGG pathway enrichment analysis, and protein-protein interaction were used to investigate the relationships between active molecules and related proteins of CVD.

HQ, MQ, DG, HFT, QN, and DH are the most commonly used herbal medicines to treat CVDs. In our work, with the help of the ADME evaluation system, 444 active ingredients were identified, 75 of which could interact with 303 direct targets by drug targeting. Recent researches have shown that some active ingredients in MHC have biological activity against CVDs, which confirm the bioinformatics data analyzed in our study and highlights the credibility of the network pharmacology system. For instance, isorhamnetin has been proven to exert cardiovascular protective effects through multiple mechanisms, including antioxidative, anti-inflammatory, and antiproliferative effects. Various pharmacological studies showed that isorhamnetin protects against cardiac hypertrophy and can exhibit positive effect on hypoxia/reoxygenation-induced injury by attenuating apoptosis and oxidative stress [25, 26]. Kaempferol can inhibit inflammatory responses and exert protective effect in LPS-induced microvascular endothelial cells [27]. Besides, the protective effect of kaempferol on heart in isoproterenol-induced heart failure has also been proven [28, 29]. Quercetin is a flavonoid that possesses pharmacological effects including antitumor, antioxidant, anti‐inflammatory, immunosuppressive, and cardiovascular protection activities [30–34]. In a recent study, it has been reported that the beta-sitosterol exerts thrombus-preventing activity by dose-dependent inhibition of thrombin in mouse model [35].

As we know, MHC probably exerts its therapeutic effect on CVD by binding and regulating particular protein targets. The analytical result of the C-T network displayed an average degree of 13 per compounds and 5.3 per target proteins, respectively. Among the 57 compounds with corresponding targets, 99 were capable of acting on more than 2 targets and 44 linked with more than 13 target proteins. The key node proteins were suggested to be important targets in the treatment of CVD. Among them, the targets prostaglandin G/H synthase 2 (PTGS2, COX-2) with the highest degree (40) and prostaglandin G/H synthase 1 (PTGS1, COX-1) with a degree of 31 can be modulated by the compounds in MHC, indicating their vital role in helping to treat CVDs. COX-2 and COX-1 are constitutively expressed in the endothelium, brain and various tissues in physiological conditions, and convert arachidonate to prostaglandin H_2_, which is responsible for production of inflammatory prostaglandins. Previous relevant studies have defined that the increased expression of COX-2 and COX-1 may contribute to the development of inflammatory diseases due to the fact that they can facilitate the transcription of several cytokines associated with disease progression. COX-2 and COX-1 have been acknowledged as classic therapeutic targets of nonsteroidal anti-inflammatory drugs (NSAIDs), which bind the site corresponding to the COX active site. Inhibition of the COX with NSAIDs acutely reduces inflammation, pain, and fever, and long-term use of these drugs reduces fatal thrombotic events, as well as the development of colon cancer and Alzheimer's disease. A recent research suggested that the inhibition of COX-2 may activate the MAPK pathway and reduce inflammatory response and improve myocardial remodeling in mice with myocardial infarction. Various activity compounds in TCM exert protective effects in the vascular or cardiac injury via the signaling or downstream processing of COX-2 [36–39]. In addition, heat shock protein 90 (HSP 90), the key enzyme in the blood coagulation system, has been identified to be the target of 31 chemicals. A recent study has shown that Hsp90 modulates cardiac ventricular hypertrophy through activating the MAPK pathway in cardiomyocytes [40]. Since HSP 90 serves as a regulator in the TGF-*β* signaling pathway, the downregulation of HSP 90 can inhibit the activation of myocardial fibroblast, which is a pathological signature of myocardial fibrosis [41]. In light of recent evidence, the activity compounds in various TCM formulas can produce therapeutic effects on atherosclerosis, cardiac injury, hypertension, thrombosis, and neurodegenerative diseases through inhibiting the expression of HSP 90 in vitro or in vivo. Thus, the bioactive ingredients from MHC interacting with COX-2, COX-1, and HSP 90 may be the key factors in the treatment of the fibroblast activation in patients with CVD.

By analyzing the GO-enriched results, we found that MHC may have certain effects in regulating cytokine-related biological processes, such as “cellular response to cytokine stimulus (GO:0071345)” and “cytokine-mediated signaling pathway (GO:0019221)”. Cytokines (such as IL-1*β*, IL-6, and TNF) derived from the vessel wall or blood have been proven to be associated with vascular risk and predictive of future cardiovascular events. Inflammatory processes and their failure to resolve is firmly established as central to the progress of cardiovascular diseases. Several emerging lines of evidence support the hypothesis that inhibition of the inflammatory processes by targeting of the cytokines might serve as an efficient system to attenuate myocardial and arterial injury, reduce disease progression, and promote healing. Our results have shown that the protective effects of MHC may be related to the inhibition of cytokines thereby suppressing chronic inflammatory process. Besides, one of the key reasons for the occurrence and development of CVD is the apoptotic process. In the present study the “regulation of apoptotic process (GO:0042981)” and “apoptotic process (GO:0006915)” are both enriched in the top ranked GO BP terms. Apoptosis is defined as a highly regulated form of cell death and can be regulated by genetic or pharmacologic interventions. Regulation of apoptosis is a hopeful choice of treatment for CVDs and disorders such as myocardial infarction, ischemia/reperfusion injury, chemotherapy cardiotoxicity, and heart failure. In the process of apoptotic, TP53, IL-6, TGF-*β*, TNF-*α*, IKBKB, and EGFR are considered to be important related proteins. The “cellular response to oxidative stress (GO:0034599)” and “cellular response to reactive oxygen species (GO:0034614)” were also enriched in our results. It is established that elevated oxidative stress could be a key factor in the development of complex biochemical, structural, and functional changes associated with CVD. Recently, different research groups have provided evidences that pharmacological approaches to counteract excessive accumulation of ROS are sufficient in the prevention or treatment of heart failure. Besides, endothelial dysfunction and vascular remodeling caused by IL-6, TGF-*β*, TNF-*α*, EGFR, and VEGFA are also some of the core features of CVD.

Our results showed that MHC integrated various signaling pathways which have been testified as accurate target pathways to modulate CVD, such as TNF, PI3K-Akt, HIF-1, FoxO, apoptosis, calcium, MAPK, Toll-like receptor, VEGF, and NF-*κ*B pathways. Most signaling pathways significantly enriched by targets were associated with multiple chronic inflammatory diseases, not merely CVD, which is consistent with the enriched GO terms in our study. Among 177 pathways, PI3K-Akt and TNF signaling as critical pathways regulate the process of apoptosis, inflammation, and oxidative stress in the development and severity of CVD. For instance, the master factors contributing to the initiation and evolution of inflammatory responses in cardiovascular tissues include the elevated level of TNF and the activation of NF-*κ*B in the TNF signaling pathway. Notably, our research indicated that the main biological function of MHC in CVD is negatively regulating the TNF signaling pathway by the interaction between bioactivity compounds and the potential targets including TNF, VCAM1, COX-2, MMP3, MMP9, IKBKB, IL-1*β*, IL-6, CCL2, CXCL2, CXCL10, caspase-3, and caspase-8. Moreover, our results also show that MHC can exert the protective effect by modulating the PI3K-Akt signaling pathway. In the cardiovascular system, a great number of studies targeting PI3K/Akt have elucidated the contribution of this pathway to cardiac and vascular function regulation both in the normal and diseased states. GSK-3*β*, AKT1, eNOS, and VEGFA, which have been screened as the targets of MHC, are considered to modulate important cellular physiological processes in the vessels, heart, and brain. The eNOS protein shows pharmacological properties by producing NO, which can rapidly diffuse across cell membranes to act as a potent paracrine mediator. The PI3K/Akt signaling pathway has been proven to be involved in the resistance response to hypoxia ischemia. It can regulate the expression of HIF-1*α*, which further modulates the expression of downstream targets that related to glucose metabolism and angiogenesis to facilitate ischemic adaptation [42]. In cardiomyocytes and smooth muscle cells, calcium fluxes are the best characterized receptor-regulated signaling events. Recent studies have proven that activation of PI3K/Akt signaling is interconnected with calcium signaling in cardiovascular system, which is emerging to make great influence in disease development [43]. Based on the results of our study, in this complex system with MHC-compounds-targets-CVDs interactions, the abovementioned active ingredients, targets, and pathways are associated with the pharmacological mechanisms of MHC in the treatment process of CVDs.

## 5. Conclusion

The current study uses a network pharmacology approach that combines active compounds, potential targets, GO, and KEGG enrichment analysis to investigate the molecular mechanism of MHC against CVDs from a systematic perspective. Among these crucial biological functions, 303 targets were identified as key active factors involved in the 177 related pathways. We found that our prediction-based results were generally consistent with previous research on pathways and diseases treated with MHC extracts. Furthermore, we can suggest more comprehensive mechanisms of therapeutic effects of MHC in terms of target proteins, pathways, and diseases than manual reviews of the literature. Nonetheless, more experimental researches were warranted to validate these hypotheses, and experimental verification of the potential effective compounds after candidate screening is needed, which will lay a foundation for further experimental research and clinical rational application of MHC.

## Figures and Tables

**Table 1:** The candidate compounds and putative target proteins of MHC.

Code	Molecule name	MW	OB (%)	DL
M1	Mairin	456.78	55.38	0.78
M2	Jaranol	314.31	50.83	0.29
M3	Hederagenin	414.79	36.91	0.75
M4	(3S,8S,9S,10R,13R,14S,17R)-10,13-Dimethyl-17-[(2R,5S)-5-propan-2-yloctan-2-yl]-2,3,4,7,8,9,11,12,14,15,16,17-dodecahydro-1H-cyclopenta[a]phenanthren-3-ol	428.82	36.23	0.78
M5	Isorhamnetin	316.28	49.6	0.31
M6	3,9-Di-O-methylnissolin	314.36	53.74	0.48
M7	5′-Hydroxyiso-muronulatol-2′,5′-di-O-glucoside	642.67	41.72	0.69
M8	7-O-Methylisomucronulatol	316.38	74.69	0.3
M9	9,10-Dimethoxypterocarpan-3-O-*β*-D-glucoside	462.49	36.74	0.92
M10	(6aR,11aR)-9,10-Dimethoxy-6a,11a-dihydro-6H-benzofurano[3,2-c]chromen-3-ol	300.33	64.26	0.42
M11	Bifendate	418.38	31.1	0.67
M12	Formononetin	268.28	69.67	0.21
M13	Isoflavanone	316.33	109.99	0.3
M14	Calycosin	284.28	47.75	0.24
M15	Kaempferol	286.25	41.88	0.24
M16	FA	441.45	68.96	0.71
M17	(3R)-3-(2-Hydroxy-3,4-dimethoxyphenyl)chroman-7-ol	302.35	67.67	0.26
M18	Isomucronulatol-7,2′-di-O-glucosiole	626.67	49.28	0.62
M19	1,7-Dihydroxy-3,9-dimethoxy pterocarpene	314.31	39.05	0.48
M20	Quercetin	302.25	46.43	0.28
M21	(2R)-5,7-Dihydroxy-2-(4-hydroxyphenyl)chroman-4-one	272.27	42.36	0.21
M22	(S)-stylopine	323.37	51.15	0.85
M23	Ziziphin_qt	472.78	66.95	0.62
M24	Icaride A	404.5	48.74	0.43
M25	Isostrychnine N-oxide (I)	352.47	35.45	0.8
M26	Isostrychnine N-oxide (II)	350.45	37.33	0.8
M27	Lokundjoside_qt	406.57	32.82	0.76
M28	Vomicine	408.54	47.56	0.65
M29	Brucine-N-oxide	410.51	49.17	0.38
M30	Isobrucine	334.45	33.58	0.8
M31	Brucine N-oxide	410.51	52.63	0.38
M32	Stigmasterol	412.77	43.83	0.76
M33	(+)-Catechin	290.29	54.83	0.24
M34	Beta-sitosterol	414.79	36.91	0.75
M35	Stigmasterol	412.77	43.83	0.76
M36	(2R,3R,3aS)-3a-Allyl-2-(1,3-benzodioxol-5-yl)-5-methoxy-3-methyl-2,3-dihydrobenzofuran-6-one	340.4	59.99	0.43
M37	Denudatin B	356.45	61.47	0.38
M38	Futokadsurin C	356.45	61.09	0.45
M39	Galgravin	372.5	57.12	0.39
M40	(2S,3S,4S,5S)-2,5-Bis(3,4-dimethoxyphenyl)-3,4-dimethyltetrahydrofuran	372.5	57.12	0.39
M41	Hancinone	340.4	39.31	0.44
M42	Isofutoquinol A	354.43	59.2	0.48
M43	(1R,5S,6R,7R,8R)-3-Allyl-6-(3,4-dimethoxyphenyl)-8-hydroxy-1-methoxy-7-methyl-4-bicyclo[3.2.1]oct-2-enone	358.47	64.65	0.35
M44	Bicyclo(3.2.1)oct-3-ene-2,8-dione, 7-(4-hydroxy-3-methoxyphenyl)-5-methoxy-6-methyl-3-(2-propenyl)-, (1R-(6-endo,7-exo))-	342.42	94.67	0.32
M45	Acetic acid [(1R,5S,6R,7R,8R)-3-allyl-6-(3,4-dimethoxyphenyl)-1-methoxy-7-methyl-4-oxo-8-bicyclo[3.2.1]oct-2-enyl] ester	400.51	59.93	0.46
M46	(2S,3S)-2-(3,4-Dimethoxyphenyl)-7-methoxy-3-methyl-2,3-dihydrobenzofuran-5-carbaldehyde	328.39	42.15	0.32
M47	Kadsurenone	356.45	54.72	0.38
M48	Kadsurin A	372.45	56.83	0.5
M49	Kadsurin B	358.47	30.55	0.46
M50	(4R)-2-Allyl-4-[(E)-2-(4-hydroxy-3-methoxyphenyl)-1-methylvinyl]-4,5-dimethoxy-1-cyclohexa-2,5-dienone	356.45	55.14	0.3
M51	Piperkadsin B	430.54	55.44	0.41
M52	Piperlactam S	295.31	40.44	0.4
M53	Stigmasterol	412.77	43.83	0.76
M54	N-Coumaroyltyramine	283.35	85.63	0.2
M55	Wallichinine	370.48	61.64	0.33
M56	Futoquinol	354.43	59.83	0.36
M57	Beta-sitosterol	414.79	36.91	0.75
M58	Acetylbullatantriol	298.47	40.21	0.18
M59	Maristeminol	322.54	30.64	0.38
M60	Eupatin	360.34	50.8	0.41
M61	Mutatochrome	552.96	48.64	0.61
M62	Physciondiglucoside	608.6	41.65	0.63
M63	Procyanidin B-5,3′-O-gallate	730.67	31.99	0.32
M64	Rhein	284.23	47.07	0.28
M65	Sennoside E_qt	524.5	50.69	0.61
M66	Torachrysone-8-O-beta-D-(6′-oxayl)-glucoside	480.46	43.02	0.74
M67	Toralactone	272.27	46.46	0.24
M68	Emodin-1-O-beta-D-glucopyranoside	432.41	44.81	0.8
M69	Sennoside D_qt	524.5	61.06	0.61
M70	Daucosterol_qt	386.73	35.89	0.7
M71	Palmidin A	510.52	32.45	0.65
M72	Beta-sitosterol	414.79	36.91	0.75
M73	Aloe-emodin	270.25	83.38	0.24
M74	Gallic acid-3-O-(6′-O-galloyl)-glucoside	484.4	30.25	0.67
M75	(-)-Catechin	290.29	49.68	0.24

## Data Availability

We have presented all our main data in the form of figures and additional file. The datasets supporting the conclusions of this article are included within the article.

## Abbreviations

MHC: Mahai capsules
CVD: Cardiovascular diseases
TCM: Traditional Chinese medicine
HQ: Hedysarum Multijugum Maxim (Huangqi)
MQ: Strychni semen (Maqianzi)
DG: Angelicae Sinensis Radix (Danggui)
HFT: Caulis Piperis Kadsurae (Haifengteng)
QN: Homalomena Occulta (Lour.) Schott (Qiannianjian)
DH: Radix Rhei Et Rhizome (Dahuang)
TCMSP: Traditional Chinese Medicine Systems Pharmacology
ADME: Absorption, distribution, metabolism, and excretion
OB: prediction of oral bioavailability
DL: Prediction of drug-likeness
GO: Gene ontology
PPI: Protein-protein interaction.

## Supplementary Materials

Supplementary Materials Table S1: targets of the 75 candidate compounds of Haima. Table S2: targets-diseases.

## References

[1] Hansson G. K. (2005). Inflammation, atherosclerosis, and coronary artery disease. *New England Journal of Medicine*.

[2] Benjamin E. J., Virani S. S., Callaway C. W. (2018). Heart disease and stroke statistics—2018 update: a report from the American heart association. *Circulation*.

[3] Ridker P. M., Everett B. M., Thuren T. (2017). Antiinflammatory therapy with canakinumab for atherosclerotic disease. *The New England Journal of Medicine*.

[4] Eikelboom J. W., Connolly S. J., Bosch J. (2017). Rivaroxaban with or without aspirin in stable cardiovascular disease. *The New England Journal of Medicine*.

[5] Cheng T. O. (2007). Cardiovascular effects of danshen. *International Journal of Cardiology*.

[6] Jiang W.-Y. (2005). Therapeutic wisdom in traditional Chinese medicine: a perspective from modern science. *Trends in Pharmacological Sciences*.

[7] Jia Y., Huang F., Zhang S., Leung S.-W. (2012). Is danshen (*Salvia miltiorrhiza*) dripping pill more effective than isosorbide dinitrate in treating angina pectoris? A systematic review of randomized controlled trials. *International Journal of Cardiology*.

[8] Sun M., Zhang J.-J., Shan J.-Z. (2009). Clinical observation of danhong injection (herbal TCM product from radix Salviae miltiorrhizae and Flos Carthami tinctorii) in the treatment of traumatic intracranial hematoma. *Phytomedicine*.

[9] He Y., Wan H., Du Y. (2012). Protective effect of danhong injection on cerebral ischemia-reperfusion injury in rats. *Journal of Ethnopharmacology*.

[10] Liu Y., Xu W., Xiong Y., Du G., Qin X. (2018). Evaluations of the effect of HuangQi against heart failure based on comprehensive echocardiography index and metabonomics. *Phytomedicine*.

[11] Gu J., Liu Y., Wu H., Li H., Liu K. (2019). Huangqi shengmai yin protects against radiation-induced cardiac fibrosis injury by regulating the TGF-*β*1/smads and MMPs. *Evidence-Based Complementary and Alternative Medicine*.

[12] Li W.-K., Wang G.-F., Wang T.-M. (2019). Protective effect of herbal medicine Huangqi decoction against chronic cholestatic liver injury by inhibiting bile acid-stimulated inflammation in DDC-induced mice. *Phytomedicine*.

[13] Wang K., Wu J., Duan X. (2017). Huangqi injection in the treatment of chronic heart failure: a systematic review and meta-analysis. *Medicine*.

[14] Zhang Y., Wu J., Guo S. (2019). The clinical efficacy and safety of the Chinese herbal medicine astragalus (Huangqi) preparation for the treatment of acute myocardial infarction: a systematic review of randomized controlled trials. *Medicine*.

[15] Hu G., Yang P., Zeng Y., Zhang S., Song J. (2018). Danggui Buxue decoction promotes angiogenesis by up-regulation of VEGFR_1/2_ expressions and down-regulation of VEGFR_1/2_ expression in myocardial infarction rat. *Journal of the Chinese Medical Association*.

[16] Liu K., Ren X.-M., You Q.-S. (2018). Ameliorative effect of dangguibuxue decoction against cyclophosphamide-induced heart injury in mice. *BioMed Research International*.

[17] Guney E., Menche J., Vidal M., Barábasi A.-L. (2016). Network-based in silico drug efficacy screening. *Nature Communications*.

[18] Kibble M., Saarinen N., Tang J., Wennerberg K., Mäkelä S., Aittokallio T. (2015). Network pharmacology applications to map the unexplored target space and therapeutic potential of natural products. *Natural Product Reports*.

[19] Lavecchia A., Cerchia C. (2016). In silico methods to address polypharmacology: current status, applications and future perspectives. *Drug Discovery Today*.

[20] Wang X., Shen Y., Wang S. (2017). PharmMapper 2017 update: a web server for potential drug target identification with a comprehensive target pharmacophore database. *Nucleic Acids Research*.

[21] Ru J., Li P., Wang J. (2014). TCMSP: a database of systems pharmacology for drug discovery from herbal medicines. *Journal of Cheminformatics*.

[22] Bindea G., Mlecnik B., Hackl H. (2009). ClueGO: a Cytoscape plug-in to decipher functionally grouped gene ontology and pathway annotation networks. *Bioinformatics*.

[23] Kuleshov M. V., Jones M. R., Rouillard A. D. (2016). Enrichr: a comprehensive gene set enrichment analysis web server 2016 update. *Nucleic Acids Research*.

[24] Szklarczyk D., Franceschini A., Wyder S. (2015). STRING v10: protein-protein interaction networks, integrated over the tree of life. *Nucleic Acids Research*.

[25] Ishola I. O., Osele M. O., Chijioke M. C., Adeyemi O. O. (2019). Isorhamnetin enhanced cortico-hippocampal learning and memory capability in mice with scopolamine-induced amnesia: role of antioxidant defense, cholinergic and BDNF signaling. *Brain Research*.

[26] Zhao T.-T., Yang T.-L., Gong L., Wu P. (2018). Isorhamnetin protects against hypoxia/reoxygenation-induced injure by attenuating apoptosis and oxidative stress in H9c2 cardiomyocytes. *Gene*.

[27] Bian Y., Liu P., Zhong J. (2019). Kaempferol inhibits multiple pathways involved in the secretion of inflammatory mediators from LPS-induced rat intestinal microvascular endothelial cells. *Molecular Medicine Reports*.

[28] Zhang L., Guo Z., Wang Y., Geng J., Han S. (2019). The protective effect of kaempferol on heart via the regulation of Nrf2, NF-*κβ*, and PI3K/Akt/GSK-3*β* signaling pathways in isoproterenol-induced heart failure in diabetic rats. *Drug Development Research*.

[29] Suchal K., Malik S., Gamad N. (2016). Kampeferol protects against oxidative stress and apoptotic damage in experimental model of isoproterenol-induced cardiac toxicity in rats. *Phytomedicine*.

[30] Chiş I. C., Baltaru D., Dumitrovici A. (2018). Protective effects of quercetin from oxidative/nitrosative stress under intermittent hypobaric hypoxia exposure in the rat’s heart. *Physiology International*.

[31] Patel R. V., Mistry B. M., Shinde S. K., Syed R., Singh V., Shin H.-S. (2018). Therapeutic potential of quercetin as a cardiovascular agent. *European Journal of Medicinal Chemistry*.

[32] Dong L. Y., Chen F., Xu M., Yao L. P., Zhang Y. J., Zhuang Y. (2018). Quercetin attenuates myocardial ischemia-reperfusion injury via downregulation of the HMGB1-TLR4-NF-*κ*B signaling pathway. *American Journal of Translational Research*.

[33] Chen X., Peng X., Luo Y. (2019). Quercetin protects cardiomyocytes against doxorubicin-induced toxicity by suppressing oxidative stress and improving mitochondrial function via 14-3-3*γ*. *Toxicology Mechanisms and Methods*.

[34] Tang J., Lu L., Liu Y. (2019). Quercetin improve ischemia/reperfusion-induced cardiomyocyte apoptosis in vitro and in vivo study via SIRT1/PGC-1*α* signaling. *Journal of Cellular Biochemistry*.

[35] Gogoi D., Pal A., Chattopadhyay P., Paul S., Deka R. C., Mukherjee A. K. (2018). First report of plant-derived *β*-sitosterol with antithrombotic, in vivo anticoagulant, and thrombus-preventing activities in a mouse model. *Journal of Natural Products*.

[36] Ahmed M. A., Kamal H. M., Taha A. M., Abd-Allateef S. F. (2018). Folic acid protects against experimental prenatal nicotine-induced cardiac injury by decreasing inflammatory changes, serum TNF and COX-2 expression. *Pathophysiology*.

[37] Al-Rashed F., Calay D., Lang M. (2018). Celecoxib exerts protective effects in the vascular endothelium via COX-2-independent activation of AMPK-CREB-Nrf2 signalling. *Scientific Reports*.

[38] Riba A., Deres L., Sumegi B., Toth K., Szabados E., Halmosi R. (2017). Cardioprotective effect of resveratrol in a postinfarction heart failure model. *Oxidative Medicine and Cellular Longevity*.

[39] Chen L., Liu P., Feng X., Ma C. (2017). Salidroside suppressing LPS-induced myocardial injury by inhibiting ROS-mediated PI3K/Akt/mTOR pathway in vitro and in vivo. *Journal of Cellular and Molecular Medicine*.

[40] Tamura S., Marunouchi T., Tanonaka K. (2019). Heat-shock protein 90 modulates cardiac ventricular hypertrophy via activation of MAPK pathway. *Journal of Molecular and Cellular Cardiology*.

[41] Cáceres R. A., Chavez T., Maestro D. (2018). Reduction of cardiac TGF*β*-mediated profibrotic events by inhibition of Hsp90 with engineered protein. *Journal of Molecular and Cellular Cardiology*.

[42] Zhang Z., Yao L., Yang J., Wang Z., Du G. (2018). PI3K/Akt and HIF-1 signaling pathway in hypoxia-ischemia (Review). *Molecular Medicine Reports*.

[43] Ghigo A., Laffargue M., Li M., Hirsch E. (2017). PI3K and calcium signaling in cardiovascular disease. *Circulation Research*.

